# Tumor hypoxia enhances non-small cell lung cancer metastasis by selectively promoting macrophage M2 polarization through the activation of ERK signaling

**DOI:** 10.18632/oncotarget.1856

**Published:** 2014-03-22

**Authors:** Jun Zhang, Ji Cao, Shenglin Ma, Rong Dong, Wen Meng, Meidan Ying, Qinjie Weng, Zibo Chen, Jian Ma, Qingxia Fang, Qiaojun He, Bo Yang

**Affiliations:** ^1^ Zhejiang Province Key Laboratory of Anti-Cancer Drug Research, Institute of Pharmacology and Toxicology, College of Pharmaceutical Sciences, Zhejiang University, Hangzhou, China; ^2^ Hangzhou First People’s Hospital, Huansha Road, Hangzhou, China; ^3^ The second Clinical Medical College, Zhejiang Chinese Medical University, Hangzhou, China; ^4^ Zhejiang Provincial People’s hospital, Shangtang Road, Hangzhou, China; ^5^ College of Materials Science and Engineering, Central South University of Forestry and Technology, Changsha, China

**Keywords:** Hypoxia, Macrophage, Metastasis, Polarization, NSCLC

## Abstract

Hypoxia is a common phenomenon occurring in the majority of human tumors and has been proved to play an important role in tumor progression. However, it remains unclear that whether the action of hypoxia on macrophages is a main driving force of hypoxia-mediated aggressive tumor behaviors. In the present study, we observe that high density of M2 macrophages is associated with metastasis in adenocarcinoma Non-Small Cell Lung Cancer (NSCLC) patients. By applying the *in vivo* hypoxia model, the results suggest that intermittent hypoxia significantly promotes the metastasis of Lewis lung carcinoma (LLC), accompanied with more CD209^+^ macrophages infiltrated in primary tumor tissue. More intriguingly, by skewing macrophages polarization away from the M1- to a tumor-promoting M2-like phenotype, hypoxia and IL-6 cooperate to enhance the LLC metastasis both *in vitro* and *in vivo*. In addition, we also demonstrate that skewing of macrophage M2 polarization by hypoxia relies substantially on activation of ERK signaling. Collectively, these observations unveil a novel tumor hypoxia concept involving the macrophage phenotype shift and provide direct evidence for lung cancer intervention through modulating the phenotype of macrophages.

## INTRODUCTION

Tumors are composed of an array of cell types, including not only cancer cells but also the non-cancer cells, and the most prominent component of these non-cancer cells are macrophages, which are often called tumor-associated macrophages (TAMs) [1]. There are two well-established polarized phenotypes, classically activated macrophages (M1) and alternatively activated macrophages (M2), both have been observed in tumor [2-3]. It is generally considered that these two types of macrophages are antagonistic and M2 macrophages function in moderating inflammatory responses, promoting angiogenesis and contributing to tissue remodeling, all of which are apparently tended to promote tumor progression [4-6]. Previous study demonstrated that NSCLC patients with a high expression of cathepsin had a poor outcome [7]. Interestingly, evidence has shown that macrophages are the primary source of cathepsin [8-9]. Thus the study on the association of macrophages infiltration and NSCLC patient survival has attracted intensive interest, but this association has rendered conflicting results. Importantly, several clues of evidence have suggested that the prognostic value of macrophages in NSCLC seems to depend on their microanatomical distribution [10]. Given that the different distribution of macrophages might resulted in interacting with the different cell types and finally determined the different polarized phenotypes of macrophages, thus the polarized phenotypes rather than the infiltration of macrophages might play a key role in the progression of NSCLC. However the macrophages activation states have been less examined in human lung carcinogenesis.

Macrophages can shift between different modes of activation and perform divergent functions [11-13], and this change is highly associated with the tumor microenvironment (TME) [14-15]. As the hallmark feature of malignant tumors, hypoxia has been proved to play an important role for TAMs infiltration in tumor tissue. Additionally, macrophages could alter their expression of several mitogenic and proangiogenic cytokines when exposed to hypoxia [16-18]. Of note, not only their numbers, but also their phenotype regulates tumorigenesis. Given that macrophages are often prominent in tumor tissues and retain a relatively immature macrophage phenotype [19-20], together with the fact that macrophages preferentially accumulate in hypoxic tumor areas [17], therefore hypoxia may also have a profound influence on the function of macrophages. However, the impact of hypoxia on the phenotype shift and functional response of macrophages is still largely unknown.

It is now generally accepted that hypoxia is linked to treatment resistance, cell proliferation, and metastatic potential, which contribute to poor prognosis [21]. Nonetheless, most of these studies only focus on the effect of hypoxia on tumor cells themselves, thus we also aimed to determine whether the action of hypoxia on TAMs is involved in hypoxia-driven tumor behaviors. In this study, we observed the infiltration of M2 macrophages was associated with the tumor metastasis in human adenocarcinoma NSCLC. Further study demonstrated hypoxia selectively promotes M2 macrophage polarization through the activation of ERK, and in turn enhances LLC metastasis and angiogenesis both *in vitro* and *in vivo*. Taken together, this study not only has attempted to characterize the effect of hypoxia on macrophage phenotype shift, but also highlights the potential of reeducation of macrophage polarization in anticancer treatment of NSCLC.

## RESULTS

### High density of M2 macrophages is associated with metastasis in adenocarcinoma NSCLC

**Figure 1:**
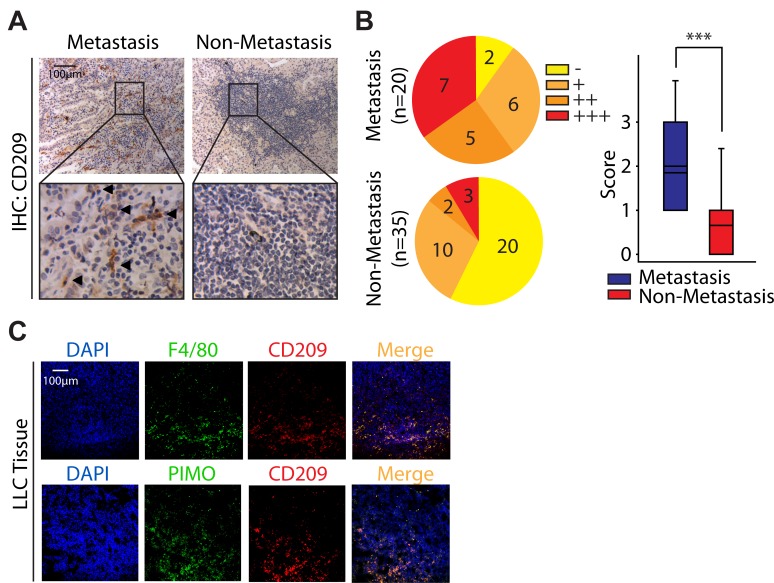
High density of M2 macrophages is associated with metastasis in adenocarcinoma NSCLC (A) Immunostaining analysis of CD209 in adenocarcinoma NSCLC. (B) The Immunostaining intensities were categorized (scored) as negative (−, 0), weak positive (+, 1), positive (++, 2), or strongly positive (+++, 3). (C) Hypoxic areas were visualized by immunolabeling of hypoxic-speciﬁc marker PIMO. F4/80 and CD209 were used as pan macrophage marker and M2 phenotype marker respectively.

Although increasing evidence suggest that M2 macrophages apparently tend to moderate inflammatory response and promote angiogenesis [4-5], all of which are seems to promote tumor progression, but their role in NSCLC is still poorly discussed. In order to address this issue, we first tried to find the clues from the GEO datasets. Interestingly, when we re-analysised microarray data (GSE1987) from 36 samples obtained from human lung tissue, the ratio of M2 macrophages rather than M1 macrophages in NSCLC was generally higher than that in normal lung tissue (Fig. S1). Encouraged by these clues, we next studied the infiltration of M2 macrophages in primary tumor tissues isolated from 55 adenocarcinoma NSCLC patients including 20 patients with metastasis. Since CD209 is believed to be a marker for M2 macrophages [22-23], the immunostaining against CD209 was performed. As illustrated in Fig. 1A and 1B, the number of CD209^+^ cells in tumors with metastasis (18/20 samples are positive for CD209^+^ cells, mean score is 1.85) was significantly higher than that in ones without metastasis (15/35 samples are positive for CD209^+^ cells, mean score is 0.66). Thus, these data indicate that the number of infiltrating M2 macrophages is correlated with metastasis in adenocarcinoma NSCLC, which is consistent with our bioinformation data drawed from the GEO dataset.

Since hypoxia is the hallmark feature of malignant tumors including NSCLC [21, 24], and our data reveal that M2 macrophages could infiltrate into the tumor tissue (Fig. 1A), we further intend to ask whether M2 macrophages are preferably localized in lung tumor hypoxic regions. In order to better characterize this issue, we established the C57BL/6 mice bearing LLC tumor model. Mice were intraperitoneally injected with PIMO, a probe extensively used for hypoxia detection[25], prior to scarified and performed further immunoﬂuorescence staining. As shown in Fig. 1C, nearly half of the macrophages (F4/80^+^) exhibited a M2 phenotype (CD209^+^). Of interest, most of these M2 macrophages (CD209^+^) were situated at hypoxic regions (PIMO^+^) (Fig. 1C), indicating tumor hypoxia might be highly associated with the M2 polarization of macrophages.

### Hypoxia enhances the metastasis of LLC accompanied with the increase of M2 macrophages in tumor tissue in vivo

**Figure 2:**
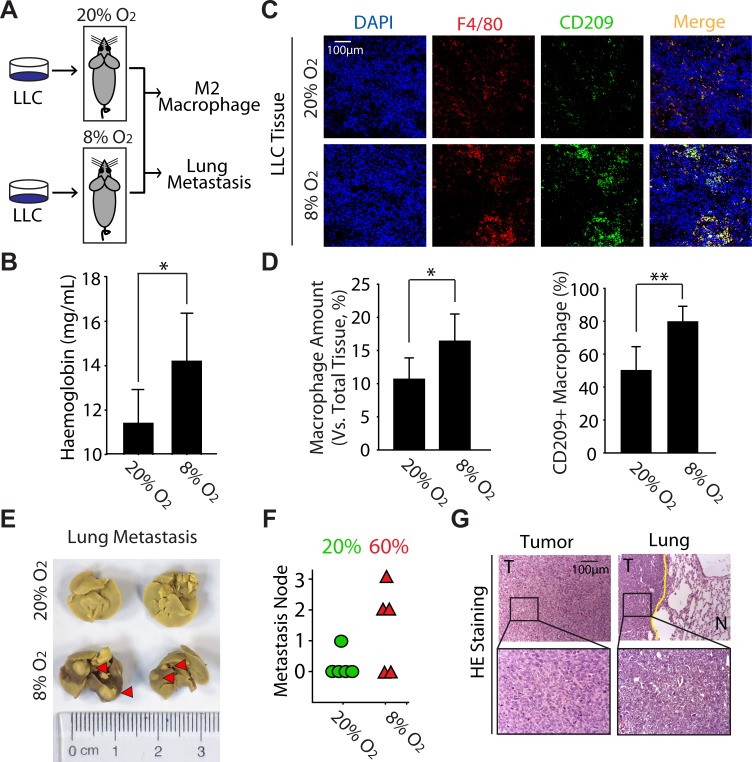
Hypoxia enhances the metastasis of LLC accompanied with the increase of M2 macrophages in tumor tissue in vivo (A) Schematic representation of experimental approach used throughout the study. (B) The level of haemoglobin was determined by blood biochemistry analyzer. (C) Colocalization of F4/80 and CD209 was visualized by immunoﬂuorescence staining. (D) Counting statistics of (C). (E) Photographs of lung metastasis (F) Quantitative analysis of lung metastasis nodules (rate). (G) LLC tumors and lung metastases were conﬁrmed by H&E staining.

Given that M2 macrophages exhibit the similar function in cancer promotion as tumor hypoxia, together with the data that M2 macrophages are preferably situated at hypoxic regions (Fig. 1C), we thus hypothesized that M2 macrophages might be involved in tumor hypoxia-driven tumorigenesis. Though there is lack of appropriate animal model for discovering the biological function of tumor hypoxia, mice receive the intermittent hypoxia via housed in a hypoxic chamber seems to meet the requests for exploring the effects of hypoxia on tumor biology [26]. Accordingly, C57BL/6 mice bearing LLC tumor were exposed to normoxia or normobaric hypoxia for 4h every day (Fig. 2A). As expected, the haemoglobin concentration was significantly increased in hypoxia-acclimated animals (Fig. 2B), which manifested our *in vivo* hypoxia model was successful [27]. Interestingly, the hypoxia exposure significantly increased the inﬁltration of macrophages (F4/80^+^) as well as the ratio of CD209^+^ macrophages (Fig. 2C, D). On the other hand, analysis of spontaneous lung metastases originating from these LLC tumors indicated that hypoxia markedly enhanced tumor metastasis to lung (from 20% to 60%) (Fig. 2E, F). Additionally, the lung metastases lesions were visualized by H&E staining (Fig. 2G). Collectively, these results provide the direct evidence to support our hypothesis that hypoxia can promote the M2 polarization of macrophages and subsequently enhance lung tumor metastasis.

### Hypoxia selectively promotes the M2 polarization of macrophages triggered by IL6

It should be emphasized that previous literatures suggest that when macrophages infiltrate into tumor, they are retained a relatively immature phenotype [19-20]. Thus the increase of CD209^+^ macrophages in hypoxia-acclimated animals (Fig. 2C, D) might mainly result from the effect of hypoxia on macrophage polarization rather than the inﬁltration of macrophages into tumor tissues (Fig. 2D). Considering this issue is rarely reported, we thus extended our study to explore the effect of hypoxia on macrophage polarization. By using the *in vitro* co-culture model of hypoxia, we first examined the inﬂuence of hypoxia on inducing experiments, in which RAW264.7 cells were incubated with (NC-CM) or without (NC) LLC-CM. As shown in Fig. S2A, comparing with NC macrophages, NC-CM macrophages expressed more CD209 and CD86 (marker for M1 macrophages). However, macrophages only exhibited a potent up-regulation of CD209 but not CD86 under hypoxic condition. These findings strongly support the idea that hypoxia plays a key role in the process of selective M2 macrophage polarization induced by lung tumor cells.

**Figure 3:**
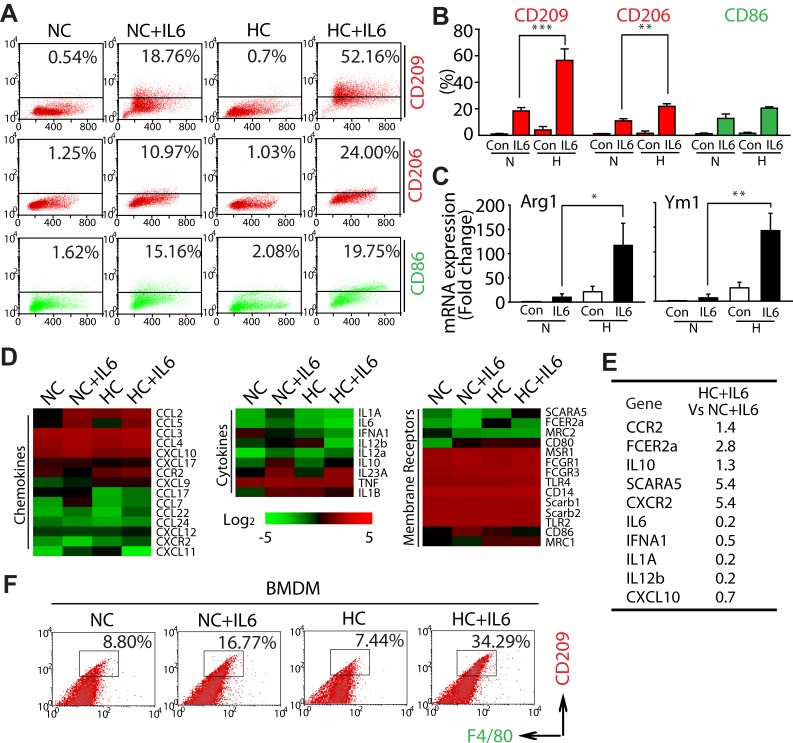
Hypoxia selectively promotes the M2 polarization of macrophages triggered by IL6 (A and B) RAW264.7 cells were exposed to normoxic or hypoxic conditions for 4 days in the presence of IL6 and then harvested for analysis of cell surface markers by ﬂow cytometry. (C) RT-PCR analysis was carried out using the Arg1, Ym1 or β-actin-speciic primers. (D) A gene microarray approach was used to compare the expression of membrane receptors, cytokines and chemokines in polarized macrophages. (E) Relative expression of M1 or M2 phenotype genes as determined by microarray analysis (HC+IL6 vs NC+IL6). (F) Flow cytometric analysis of the percentage of M2 BMDMs.

Because *A*) IL6 has been implicated in NSCLC poor progression basing on clinical reports [28], *B*) immunostaining derived from LLC tumor tissue showed IL6 accumulated in F4/80^+^ macrophages areas (Fig. S2B), and *C*) anti-IL6 antibody impaired the increase of M2 macrophages (CD209^+^) induced by LLC-CM under hypoxic condition (Fig. S2C), we were thus encouraged to gain specific insight into the phenotype shift of macrophages during hypoxia by applying the IL6. Comparing with treatment with IL6 alone in RAW264.7 cells (NC+IL6), co-treatment with IL6 and hypoxia (HC+IL6) elevated the percentage of both CD209^+^ and CD206^+^ (another marker for M2 macrophages) cells, but the percentage of M1 macrophages were not increased significantly (Fig. 3A, B). Intriguingly, the similar phenomenon was also achieved in IL4 or IL13-mediated macrophage polarization (Fig. S3).

In order to further clarify this issue, the mRNA expression of M2 phenotype markers was also examined. As shown in Fig. 3C, the mRNA expression of ARG1 and YM1 was significantly higher in HC+IL6 macrophages compared with that in the matched NC+IL6 macrophages. Since M2 macrophages express factors involved in immunosuppressive or trophic properties [1, 29-32], a gene microarray approach was used to compare the expression of receptors, cytokines and chemokines in polarized macrophages. As shown in Fig. 3D and 3E, comparing with NC+IL6 macrophages, HC+IL6 macrophages expressed low levels of M1 phenotype genes (IL12, IL6, IFNa1, IL1a and CXCL10) and high levels of the M2 phenotype genes (FCER2a, SCARA5, IL10, CCR2 and CXCR2). We subsequently extended our study into a primary macrophages model basing on the generation of BMDMs [33]. Once again, IL6-treated BMDMs (F4/80^+^) exposed to hypoxia expressed higher levels of CD209 compared with the ones exposed to normoxia (Fig. 3F). In summary, these data mentioned above clearly demonstrate that hypoxia selectively promotes the M2 polarization of macrophages triggered by IL6.

### Hypoxia-promoted M2 macrophages enhance the progression of LLC both in vitro and in vivo

**Figure 4:**
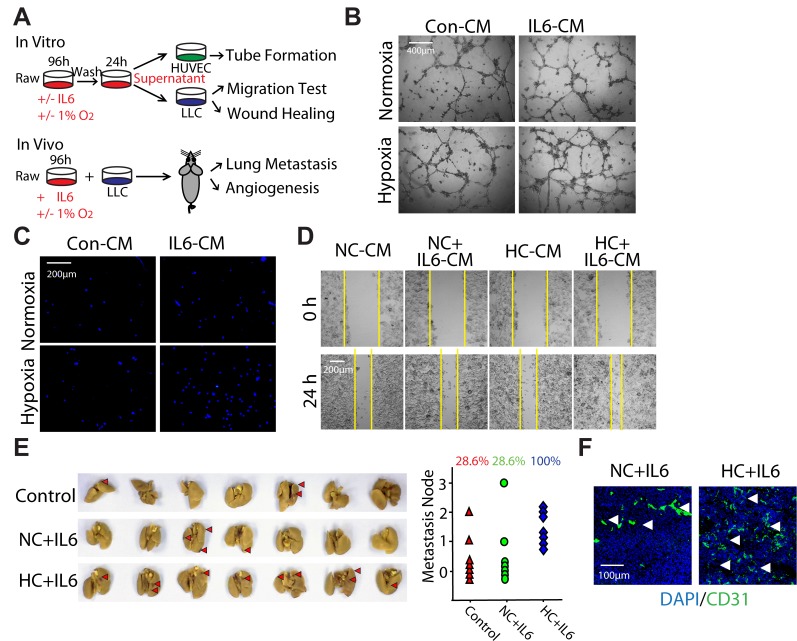
Hypoxia-promoted M2 macrophages enhance the progression of LLC both in vitro and in vivo (A) Schematic representation of experimental approach used throughout the study. (B) HUVEC cells were formed capillary-like structures in the presence of indicated macrophage-CMs. (C) LLC cells were incubated with indicated macrophage-CMs using a 24-well transwell chamber, and the invasive cells were stained with DAPI. (D) A wound-healing assay was used to examine the effect of activated macrophages on LLC cells migration. (E) LLC cells mixed with different activated macrophages were injected subcutaneouly into the armpit of C57BL/6 mice. Mice were assigned to 3 groups: control group, NC+IL6 group and HC+IL6 group. (n=7 per group). Photographs of lung metastasis and quantitative analysis of lung metastasis nodules (rate). (F) Representative photomicrographs showing immunofluorescence staining for CD31 in tumor sections

On the basis of the findings that hypoxia could selectively promote M2 macrophage polarization triggered by IL6 (Fig. 3), we prompted to validate the biological function of hypoxia-promoted M2 macrophages on the promotion of LLC tumor both *in vitro* and *in vivo* as outlined schematically in Fig. 4A. HUVEC cells were seeded in a 96-well plate pre-coated with matrigel and formed capillary-like structures in the presence of different supernatant of macrophages. The network of tube-like structures in HC+IL6 group was more extensive than that in other three groups (Fig. 4B). We next examined whether hypoxia-promoted M2 macrophages had the ability to enhance LLC cells migration. To address this question, transwell assay and wound-healing assay were performed. After 24h of culture in CM, the migration of LLC cells in HC+IL6 group was highest among the four groups (Fig. 4C, D).

To determine whether hypoxia-promoted M2 macrophages stimulate the progression of LLC *in vivo*, we injected LLC cells mixed with macrophages into the armpit of C57BL/6 mice. As shown in Fig. 4E, the metastasis rate of LLC transplantation tumors increased from 28.6% to 100% after co-injecting with hypoxia-promoted M2 macrophages. Additionally, Co-inoculation of hypoxia-promoted M2 macrophages increased the proportion of CD31-positive cells, implying more angiogenesis were formed in HC+IL6 group (Fig. 4H). Once again, these results, in line with our *in vitro* results, demonstrate that hypoxia-promoted M2 macrophages could enhance the progression of LLC both *in vitro* and *in vivo*, and further support our hypothesis that hypoxia-promoted M2 macrophages play a critical role in hypoxia-triggered NSCLC tumorgenesis.

### MAPKs might be involved in the selectively promotion of M2 Macrophage polarization under hypoxic conditions

**Figure 5:**
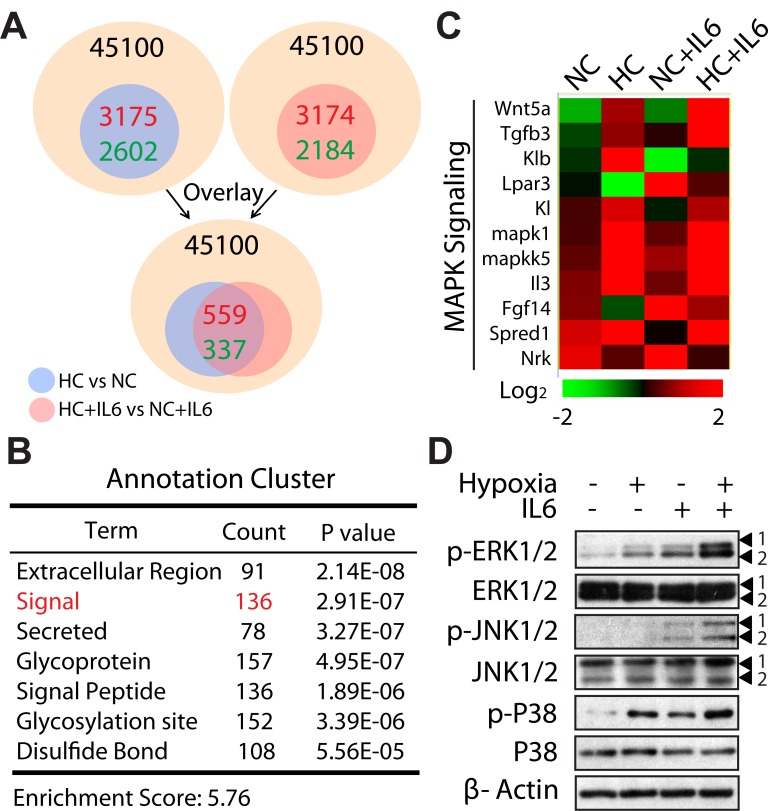
MAPK cascade is operative in M2 macrophage polarization under hypoxic conditions (A) Schematic representation of comparing gene expression proﬁles in polarized macrophages (2-fold difference, HC vs NC; HC+IL6 vs NC+IL6) and overlay analysis. (B) Hierarchical clustering of the above-mention genes. (C) Heat map of genes correlated with MAPK signaling. (D) RAW267.4 cells were treated for 24 h in normoxia or hypoxia in the presence or absence of IL6. Proteins were detected by western-blot analysis.

Next, we used microarray analysis to gain speciﬁc insight into the mechanism of M2 macrophage polarization during hypoxia. Genes whose expression differed from ≥2 fold in NC macrophages vs HC macrophages were identiﬁed, with 3,175 genes up-regulated and 2, 602 genes down-regulated. Similarly, we performed to compare gene expression proﬁles of NC+IL6 macrophages and HC+IL6 macrophages, among 45,100 total genes, 5,358 genes were determined to be altered (2-fold difference, up: 3,174 genes, down: 2,184 genes). Following overlay analysis, about 900 genes were found and then clustered (Fig. 5A). Of these, a cluster highlighted by GO analysis was associated with signal pathway (136 genes) (Fig. 5B). More interestingly, in this cluster, a relevant percentage of these genes was correlated with mitogen-activated protein kinase (MAPK) signaling (Fig. 5C). The MAPK family consists of three parallel signal transduction modules converging on the serine/threonine kinases JNK, p38, and ERK [34]. Since JNK, p38, and ERK can only exert their activity after phosphorylation, we next monitored the expression level and phosphorylation status of these kinases. Consistent with microarray data, IL6 stimulation resulted in increased phosphorylation of ERK and JNK as well as P38, and the effects were amplified in the presence of hypoxia (Fig. 5D). Therefore, these results suggest that MAPKs might be involved in the selectively promotion of M2 macrophage polarization under hypoxic conditions.

### Hypoxia promotes M2 macrophages polarization and tumorigenesis by activating ERK signaling

**Figure 6:**
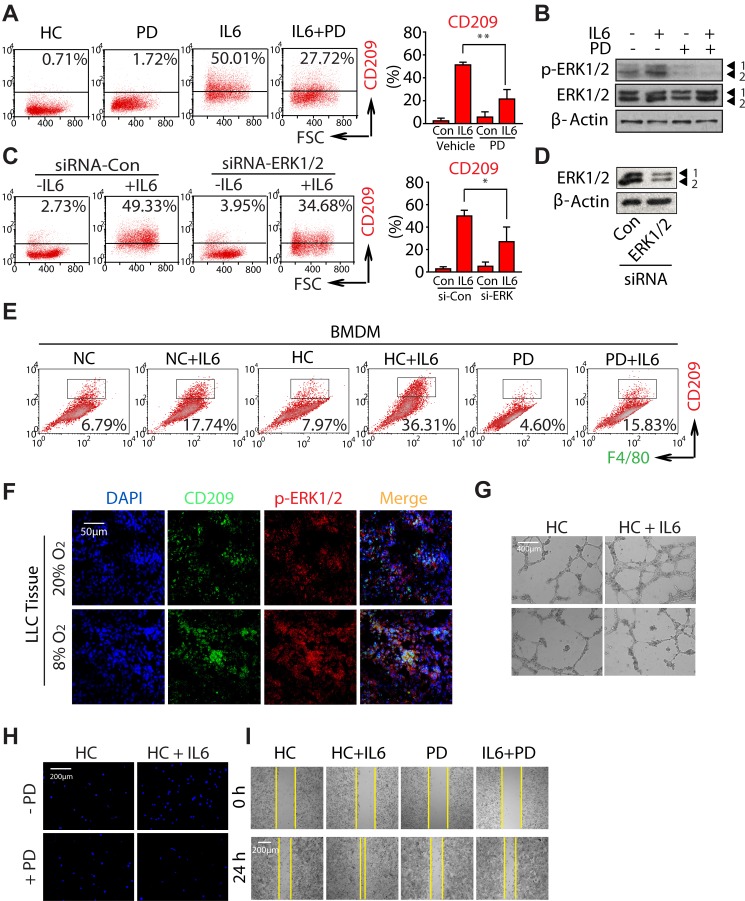
Hypoxia promotes M2 macrophages polarization and tumorigenesis by activating ERK signaling RAW264.7 cells were incubated with 20μM PD in the presence or absence of IL6 under hypoxic conditions. (A) Flow cytometric analysis was performed to analyze the percentage of CD209^+^ cells. (B) Proteins were detected by western-blot analysis. (C) RAW264.7 cells were transfected with siRNAs specifically targeting ERK1/2 or control siRNAs, as described in the *Materials and Methods*. (C) Flow cytometric analysis of the percentage of CD209^+^ cells. (D) Proteins were detected by western-blot analysis using specific antibodies. (E) BMDMs were exposed to PD in the presence or absence of IL6. Flow cytometric analysis was used to analyze the percentages of F4/80^+^CD209^+^ cells. (F) Colocalization of p-ERK and CD209 was visualized by immunoﬂuorescence staining (G) PD inhibits HUVEC cells tube formation. (H and I) PD inhibits LLC cells migration.

In an attempt to explore which pathway of MAPKs is involved in the progression of M2 macrophage polarization, we utilized the selective inhibitors: PD98059 (PD), SP600125 and SB203580 (well-known MEK, JNK, and p38 inhibitors, respectively). As shown in Fig. 6A and Fig. S4A, only PD treatment significantly attenuated hypoxia and IL6-induced CD209 expression, while SP600125 and SB203580 did not have inhibitory effects, even though the concentration we chose was sufficient to inhibit the activation of each pathway induced by hypoxia (Fig. 6B and Fig. S4B). In order to directly address the contribution of ERK pathway to the M2 macrophage polarization during hypoxia, we knocked down ERK1/2 expression using specific siRNAs (Fig. 6D). As expected, ERK1/2-targeting siRNA significantly decreased the number of CD209^+^ macrophages induced by hypoxia (Fig. 6C). Moreover, the similar results were also achieved in the BMDMs polarization model after treating with PD (Fig. 6E). On the other hand, immunofluorescence derived from LLC tumor tissue showed CD209 colocalized with p-ERK1/2, and the infiltration of CD209^+^ macrophages was increased as well as the expression of p-ERK1/2 in hypoxia-acclimated animals (Fig. 6F). Take together, all of these findings indicate that ERK signaling plays a key role in hypoxia-induced M2 macrophage polarization.

Of note, comparing with cancer cells, the activation of ERK1/2 was predominately observed in CD209^+^ macrophages (Fig. 6F). Since evidence raises the interesting possibility of targeting macrophage polarization as an innovative therapeutic strategy [30, 35], these findings further propose the possibility that reeducation macrophages could be achieved through the modulation of ERK signaling. Subsequently, we studied the effect of PD on HC+IL6 macrophages-induced tumor malignant transformation. Given that the concentration of compound used for macrophages-targeted therapy should not induce cancer cell death directly, parallel studies of cell viability were first performed (Fig. S5A). After treating LLC cells with PD, no significant alteration of cell viability was observed relative to the untreated control group. Next, in order to address whether the angiogenesis-promoting effect of HC+IL6 macrophages can be abolished by blocking ERK activation with PD, a matrigel tube formation assay was performed. As shown in Fig. 6G, PD impaired the tube formation caused by HC+IL6 macrophages. In addition, the transwell assay and wound-healing assay were also performed. As expected, the migration-promoting effect of HC+IL6 macrophages on LLC cells was blocked by PD (Fig. 6H, I), but the migration capability was unaffected when PD directly exposed to LLC (Fig. S5B). Collectively, these data suggest targeting ERK signaling is a powerful way to modulate the macrophage phenotype, and could be served as a promising lung cancer therapeutic strategy.

## DISCUSSION

Recent evidence has shown that hypoxia is relevant in macrophage activation and function [17]. For example, macrophages migrate to and aggregate in hypoxic areas because hypoxic tumors secrete higher amounts of chemotactic factors [11]. However, the potential of hypoxia to alter macrophage phenotype and function has been rarely studied. By using an *in vitro* hypoxia model, we provided the evidence that hypoxia could selectively promote M2 macrophage polarization triggered by IL6 (Fig. 3). Importantly, these hypoxia and IL6-induced M2 macrophages were more effective at promoting LLC tumor metastasis (Fig. 4), suggesting the tumor-promoting effect of macrophages was also enhanced during the tumor hypoxia. Given that our data also demonstrate hypoxia enhances LLC metastasis accompanied with the increase of M2 macrophages *in vivo* (Fig. 2), these results might further describe an alternative way of hypoxia to aggravate tumor metastasis via skewing macrophage polarization into an M2 phenotype rather than its direct effects on the inherent adhesive and invasive ability of tumor cells.

Colonization of tumor cells injected through subcutaneous (spontaneous) or intravenous is widely used as a model for detecting metastasis. However, comparing to intravenous injection model, spontaneous metastasis model involves a more comprehensive process that primary transplanted tumor is allowed to grow and spread from its primary site to other parts of the body [36]. Because of the development of hypoxia and the infiltration of macrophages in the primary tumor, spontaneous metastasis model is a more reasonable assay to explore the role of hypoxia-driven M2 macrophages in tumor metastasis. In our case, results revealed that LLC cells exhibited a weak spontaneous metastatic ability (about 20%-30%). However, the metastasis rate was strongly enhanced after hypoxia treatment (about 60%) or co-inoculation with HC+IL6 macrophages (almost 100%). Therefore, though only several metastatic nodes were observed, the incidences of lung metastasis fully proved that hypoxia-driven M2 macrophages play a crucial role in the promotion of metastasis.

Understanding the mechanism of M2 macrophage polarization during tumor hypoxia is important. Studies have revealed that HIF-1α leads to an M1-like macrophage polarization, whereas HIF-2α skews macrophage toward a M2 phenotype [37-39]. TAMs normally appear to exhibit a mixed M1/M2 polarized phenotype related to the contradictory contribution of HIF factors [40]. In this context, attempts were therefore undertaken to determine whether HIF activity was responsible for the shift between polarization states in our study. RAW264.7 cells were pretreated with acriflavine (ACF, a HIF dimerization inhibitor) [41] before the addition of IL-6 and the induction of hypoxic challenge to block HIF transcriptional activity. As expected, we detected decreased expression of BNIP3 (a classic HIF-1 target gene), indicating that ACF was active in these cells (FigS6A). As shown in FigS6B, mono-treatment with IL6 induced 52.16% CD209^+^ cells, and the combination of IL6 and ACF led to 62.07% CD209^+^ cells. Notably, mono-treatment with ACF resulted in 13.30% CD209^+^ cells, suggesting the effect of co-treatment was the addition of all individual effect. Such ﬁndings indicates that HIF activity did not participate in the M2 macrphage polarization in response to IL-6 plus hypoxia.

IL-6 has been implicated as an important activator of oncogenic transcription factor STAT3 [42]. STAT3 activation is revealed to contribute to the M2 polarization of Mɸs [43]. Indeed, our current study (FigS6C) similarly indicates that STAT3 signaling pathway was highly activated in the presence of IL-6. However, phospho-STAT3 induced by IL-6 was unaffected after hypoxia treatment. One can conclude that STAT3 activation is not a critical event for maintaining the malignant M2 phenotype of HC+IL6 macrophages.

IL-6 does not only lead to the activation of the STAT signalling, but also to the induction of the MAPK cascade [44]. Data from microarray assay and western blot analysis suggest that MAPK cascade is operative in our experimental model (Fig. 5). On the basis of specific inhibitors and siRNA results (Fig. 6), we proposed that hypoxia could augment IL6-induced ERK signaling and thus lead to an M2 macrophage polarization. Here, the important influence of ERK signaling pathway on macrophage phenotype shift and functional response is established, but several key questions involving mechanisms responsible for these phenomena need yet to be answered. These include how hypoxia amplifys ERK activation and what transcription factor(s) is controlled by ERK cascade which accounts for macrophage polarization. In this regard, further studies will be undertaken to clarify these considerations.”

While all three MAPKs, ERK, JNK and p38, are activated under hypoxia conditions, only the inhibition of ERK activation can block hypoxia-induced M2 macrophage polarization (Fig. 6). The finding that co-localization of CD209 and p-ERK1/2 in LLC tumor tissue demonstrated that ERK of CD209^+^ macrophages was relatively active comparing to tumor cells (Fig. 6F). More importantly, we observed that PD has negligible effect on the proliferation and migration of LLC cells *in vitro* (Fig. S5), suggesting that PD could be used to suppress tumor metastasis by targeting TAMs but not by directly affecting tumor cells. Of note, several small-molecular inhibitors of ERK have been developed and are currently being tested in clinical trials [45]. Thus, the present results not only underscore the important influence of ERK signaling pathway on macrophage phenotype shift, but also provide further support for ERK-blockage strategies for reeducation of macrophage polarization and efficiently suppressing tumorigenesis.

IL6 is a well-known pleiotropic, pro-inflammatory cytokine which plays a role in immune response, cell differentiation and growth [46-47]. Previous studies have reported that IL6 is over-expressed in TME and acts to generate M2 macrophages [48-49]. Consistently, we observed that IL6 accumulated in F4/80^+^ macrophages areas of LLC tumor (Fig. S2B) and induced CD209 (CD206) expression in NC+IL6 macrophages (Fig. 3A). Interestingly, we also found that NC+IL6 macrophages expressed high levels of CD86 and exhibited no obvious tumor-promoting M2 phenotype function (Fig. 4). However, when exposed to hypoxia, IL6 shaped more macrophages differentiation into M2-like cells and functioned in promoting tumor angiogenesis and metastasis. Our works support the hypothesis that TAMs are predisposed to have M2 function due to tumor released factors and the development of hypoxia [50]. Importantly, besides IL6, the similar results were also observed in macrophages treated with IL4 or IL13 (Fig. S3), implying hypoxia is critical for the switch that maintains the promalignancy phenotype (M2) of macrophages. Therefore, although anti-interleukin therapies exhibit the anticancer activity, targeting the hypoxia-driven macrophage education might be a more rational strategy for cancer treatment.

In summary, we identify tumor hypoxia selectively promotes M2 macrophage polarization through the activation of ERK, and in turn enhances the NSCLC metastasis. These observations highlight a novel tumor hypoxia concept involving the phenotype shift of macrophages and open new insights into improving the efficiency of lung cancer treatment by TAMs’ reeducation.

## MATERIALS AND METHODS

### Materials

Recombinant IL6 was purchased from R&D Technology. PD98059 was from Sigma-Aldrich. Recombinant IL4 and IL13 were purchased from Peprotech. SP600125 and SB203580 were obtained from Selleck Chemicals. The following primary antibodies to p-ERK (Thr202/Tyr204), ERK, p-JNK (Thr183/Tyr185), JNK, p-p38 (Thr180/Tyr182), p38 were from Cell Signaling.

### Cell lines and cell culture

RAW264.7 cells and LLC cells were obtained from the Cell Bank of the China Science Academy (Shanghai, China), and maintained in DMEM containing 10% FBS. Moderately hypoxic conditions (1% O_2_) were achieved by putting cells in a hypoxia incubator filled with a mixture of 1% O_2_, 5% CO_2_ and 94 % N_2_.

### Preparation of bone marrow-derived macrophages (BMDMs)

Bone marrow isolation was performed as described previously [51]. The bone marrow cells differentiated into BMDMs with M-CSF (Cell Signaling). After a three days incubation, BMDMs were rinsed with DMEM to remove nonadherent cells and then cultured with 25 ng/ml IL6 for additional 5 days.

### Conditioned medium (CM) preparation

Macrophage polarization was obtained by culturing cells with 25 ng/ml IL6 under normoxia or hypoxia for 4 days. Where indicated, chemical inhibitors were added during macrophage polarization. Different polarized macrophages were incubated in serum free medium for 24 h, after which culture supernatants were collected as CM. CM derived from LLC were obtained and used as described previously [52]. CM was centrifuged at 2000 rpm to separate out the debris and stored at -80□. For stimulation with LLC-CM, RAW264.7 cells were supplemented with LLC-CM to a ﬁnal concentration of 50% (vol/vol). In neutralization experiments, LLC-CM was incubated with or without anti-IL6 antibody for 1h before adding to the RAW264.7 cells.

### Biopsy specimen

This study was conducted with a total of 55 paraffin-embedded human lung adenocarcinoma specimens that were histo-pathologically diagnosed at Zhejiang Provincial People’s Hospital and Hangzhou First People’s Hospital from 2010 to 2012.

### Flow cytometry

Samples were incubated with PE-CD86, FITC-CD206 (Biolegend), PE-conjugated CD209 or F4/80 antibody (eBioscience) according to the manufacturers’ instructions. Fluorescent conjugated with Alexa Fluor 488 (Invitrogen) was used as a secondary antibody. For each sample at least 1×10^4^ cells should be analyzed.

### Reverse transcription-PCR

The quantitative real-time RT-PCR analysis was performed by TAKARA SYBR Premix EXTaqTM. The reaction mixtures containing SYBR Green were composed following the manufacturer’s protocol. The sequences of the primers used for the quantitative RT-PCR were as follows: ARG1: 5′-CACTCCCCTGACAACCAGCT-3′ and 5′-AGGACACAGGTTGCCCATG-3′; YM1, 5′-TCTCTACTCCTCAGAACCGTCAGA-3′ and 5′-GATGTTTGTCCTTAGGAGGGCTTC-3′; ACTIN, 5′-GGTCATCACTATTGGCAACG-3′ and 5′-ACGGATGTCAACGTCACACT-3′.

### Cell transfection

The siRNA sequence was duplexes produced by Genepharma, Co. (Shanghai, China). The sequences of siRNAs used were as follows: ERK1: AATGTTATAGGCATCCGAGAC; ERK2: AAAGTTCGAGTTGCTATCAAG. The transfection was performed using jetPRIME (Polyplus Transfection) according to the manufacturer’s recommendations.

### Migration assay

LLC cells Migration assay was performed in a transwell Boyden Chamber. Cell suspension (2×10^5^ cells/ml) was placed in the upper chamber. The lower compartment contained 0.6 ml of CM. After 24-hour incubation at 37 °C, cells were fixed with 70% EtOH and then stained with DAPI. The stained cells were subsequently photographed.

### Wound healing assay

Seed LLC cells in 24-well plates and culture until 70-80% confluent. Using a pipette tip make a straight scratch, formation an artificial wound. Treatment with CM, the migration of cells across this artificial wound was assessed.

### Tube Formation assay

HUVEC cells (2×10^4^ cells/well) were seeded into a 96-well plate that had been pre-coated with 50 μl Matrigel (BD Transduction Laboratories, San Jose, USA) and cultured with CM. Formation of tube-like structures was monitored by microscopic observation.

### Immunofluorescence

Tumor hypoxia was studied by intraperitoneal injection of 60 mg/kg pimonidazole (PIMO) hydrochloride (Hypoxyprobe™-1 Kit, Chemicon) 1 h before tumor harvest. Cryostat sections were ﬁxed and permeated. PIMO, F4/80, CD209 and CD31 (Abcam, Cambridge) antibodies were used, followed by Alexa Fluor 488 or 594. Nuclei were visualized by staining DAPI.

### Immunohistochemical (DAB)

Paraffin-embedded tissue sections were dewaxed, rehydrated, and subjected to microwave with PH 9.0 Tris-EDTA buffer for CD209 staining. Then Histostain-Plus Kit was used by following the manufacturer’s instructions.

### cDNA Microarray

The RNA samples of were hybridized using Affymetrix Mouse Genome 430A 2.0 Oligonucleotide Microarrays in the Shanghai Biotechnology Corporation (Shanghai, China). After scanning, hybridization signals were collected for further analysis. The entire microarray dataset is available at Gene Expression Omnibus database under accession no. GSE50073.

### LLC spontaneous metastasis model

C57BL/6 female mice (National Rodent Laboratory Animal Resource, Shanghai, China), 4–5 weeks of age, were used for all experiments. All animal experiments were carried out in accordance with the Institutional Animal Use and Care Committee.

Co-inoculated tumor model: LLC cells (1×10^6^) mixed with or without conditioned macrophages (1:3) were implanted subcutaneouly into the armpit of mice for 4 weeks as described previously [53]. Mice were randomly chosen and assigned to 3 groups (7 animals per condition) based on the difference of macrophages co-inoculated with LLC cells: control group (LLC cells), NC+IL6 group (RAW264.7_IL6_ + LLC cells) and HC+IL6 group (RAW264.7_IL6+hypoxia_ + LLC cells).

In Vivo Hypoxia Model: Tumors were established by injection of LLC cells (1×10^6^) into the armpit of mice. After 3 days of injection, mice were exposed to normobaric hypoxia (8% O_2_, 92% N_2_) or room air for 4h every day (5 animals per condition). Mice were killed 5 weeks later.

At the termination of experiment, tumor tissues were harvested and weighted, and the presence of lung metastases was determined grossly and microscopically.

### Statistical analysis

Data were presented as means ± SD, and the significance of the differences between the values of the groups was determined with Student’s t-test. Differences were considered significant at P ≤ 0.05.

## SUPPLEMENTARY FIGURE AND TABLE


