# Supplementary material for: Tumor hypoxia enhances non-small cell lung cancer metastasis by selectively promoting macrophage M2 polarization through the activation of ERK signaling
Source: Oncotarget. 2014 Mar 22;5(20):9664–77. doi: 10.18632/oncotarget.1856 (PMC4259428; doi:10.18632/oncotarget.1856)
Supplement: Supplementary file 1 [file oncotarget-05-9664-s001.pdf]

Tumor hypoxia enhances Non-Small Cell Lung Cancer metastasis by selectively promoting macrophage M2 polarization through the activation of ERK signaling – Zhang et al

ID	Description	CD206 relative expression		CD86 relative expression	
2000N_1	Commercial normal lung RNA	0.360		3.433	
2000N_2	Commercial normal lung RNA	1.232		6.971	
2059N	Normal tissue adjacent to sample 2059T	3.630		14.337	
2065N	Normal tissue adjacent to sample 2065T	1.446		9.020	
2073N	Normal tissue adjacent to sample 2073T	2.364	2.008	30.898	12.572
2075N	Normal tissue adjacent to sample 2075T	2.725		15.360	
2079N	Normal tissue adjacent to sample 2079T	0.855		9.219	
2091N	Normal tissue adjacent to sample 2091T	4.845		21.090	
2092N	Normal tissue adjacent to sample 2092T	0.612		2.821	
2002T	Adenocarcinoma. Male. Stage 1	17.165		26.655	
2009T	Squamous Cell Carcinoma. Male. Stage 1.	0.769		6.035	
2011T	Squamous Cell Carcinoma. Femeale. Stage 1.	6.275		12.970	
2014T	Squamous Cell Carcinoma. Male. Stage 1.	3.245		28.395	
2062T	Adenocarcinoma. Male. Stage 1.	2.840		34.225	
2076T	Squamous Cell Carcinoma. Male. Stage 1.	3.595	3.995	11.830	18.670
2081T	Squamous Cell Carcinoma. Male. Stage 1.	2.441		5.841	
2083T	Squamous Cell Carcinoma. Female. Stage 1.	4.260		16.105	
2087T	Squamous Cell Carcinoma. Male. Stage 1.	1.015		5.402	
2090T	AdenoSquamous. Male. Stage 1.	2.770		22.270	
2093T	Adenocarcinoma. Female. Stage 1.	0.656		8.209	
2097T	Squamous Cell Carcinoma. Male. Stage 1.	2.910		46.110	
2001T	Squamous Cell Carcinoma. Femeale. Stage 2.	1.286		9.720	
2010T	Adenocarcinoma . Femeale. Stage 2.	1.197		13.501	
2022T	Adenocarcinoma. Male. Stage 2.	5.375		20.760	
2073T	Squamous Cell Carcinoma. Male. Stage 2.	2.282	3.697	19.216	18.477
2075T	Squamous Cell Carcinoma. Male. Stage 2.	4.050		17.693	
2099T	Squamous Cell Carcinoma. Male. Stage 2.	2.220		29.860	
2068T	Squamous Cell Carcinoma. Male. Stage 2.	9.470		18.585	
2017T	Adenocarcinoma. Femeale. Stage 3.	1.514		8.566	
2020T	Adenocarcinoma. Femeale. Stage 3.	1.660		11.514	
2069T	Squamous Cell Carcinoma. Male. Stage 3.	16.540	4.440	21.620	15.222
2079T	Squamous Cell Carcinoma. Male. Stage 3.	2.997		18.997	
2091T	Squamous Cell Carcinoma. Male. Stage 3.	0.975		6.523	
2092T	Squamous Cell Carcinoma. Male. Stage 3.	2.955		24.115	

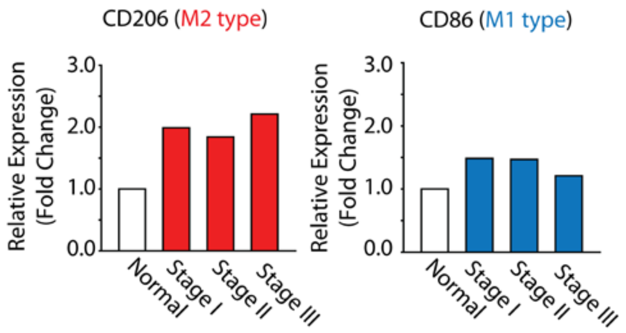

Figure S1. M2 macrophages are associated with the poor progression of NSCLC. The expression of CD206 and CD86 was analysed in microarray data (GSE1987).

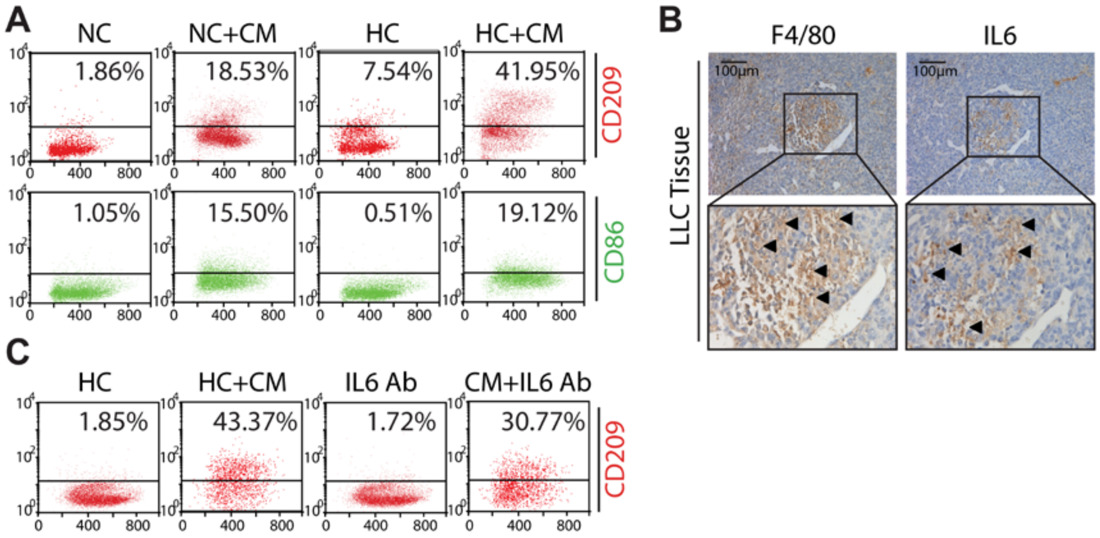

Figure S2. IL6 is a main factor involved in macrophage polarization in the LLC-CM. (A) RAW264.7 cells were incubated without or with the LLC-CM for 72 h under normoxic/hypoxic conditions, and flow cytometric analysis was performed to analyze the expression of CD209 and CD86. (B) Tumor-infiltrating macrophages were visualized by F4/80 staining. IL6 was detected by immunohistochemical staining in the same LLC tumor section. (C) RAW264.7 cells were maintained in LLC-CM with or without neutralizing IL6 antibody. The percentage of CD209^{+} macrophages was detected at 72 h.

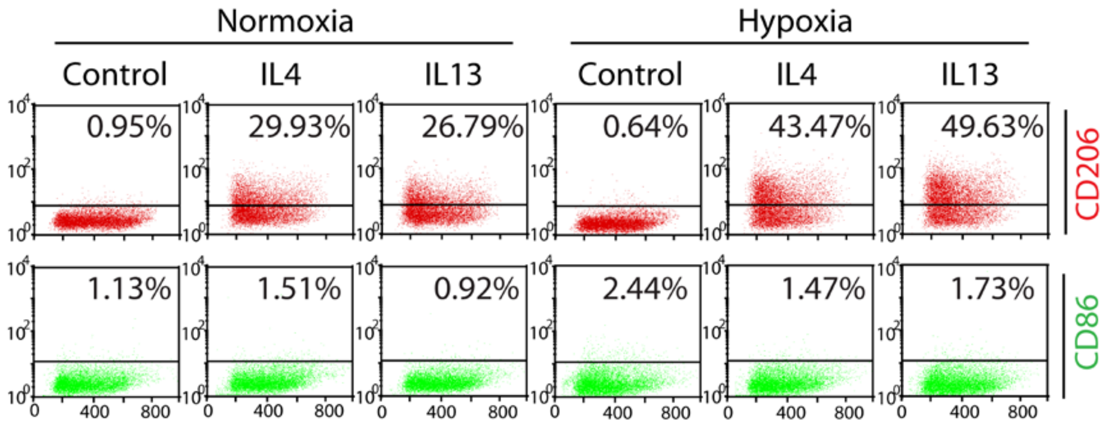

Figure S3. Effects of hypoxia on IL4 (IL13)-induced macrophage polarization. RAW264.7 cells were exposed to normoxia or hypoxia, in the presence or absence of 10μg/ml IL4 (IL13). Flow cytometric analysis was performed to analyze the expression of CD206 and CD86.

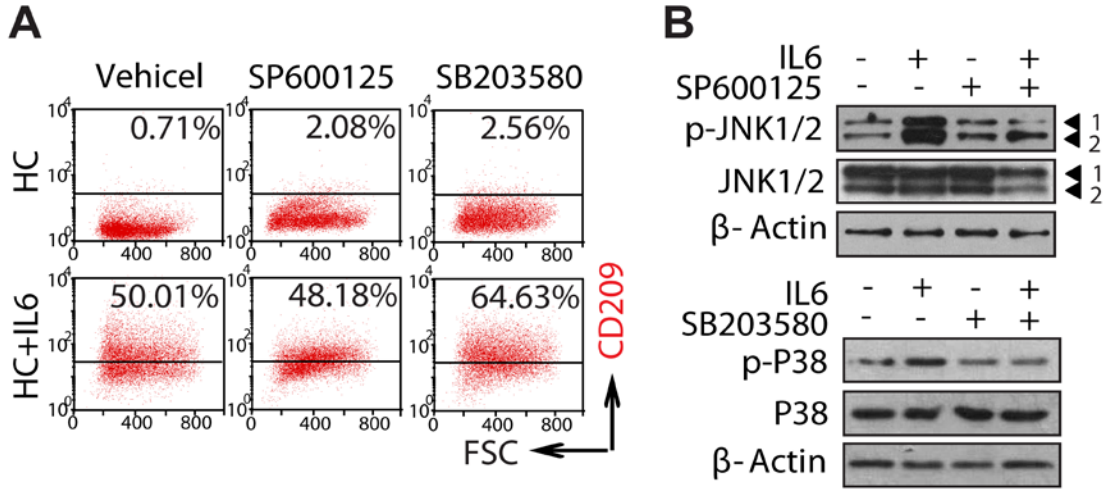

Figure S4. JNK and p38 MAPK are not involved in hypoxia-induced M2 macrophage polarization. RAW264.7 cells were incubated with SP600125 (10μM) and SB203580 (10μM), respectively, in the presence or absence of IL6. (A) Flow cytometric analysis was performed to analyze the percentage of $CD209^{+}$ cells. (B) Proteins were detected by western-blot analysis.

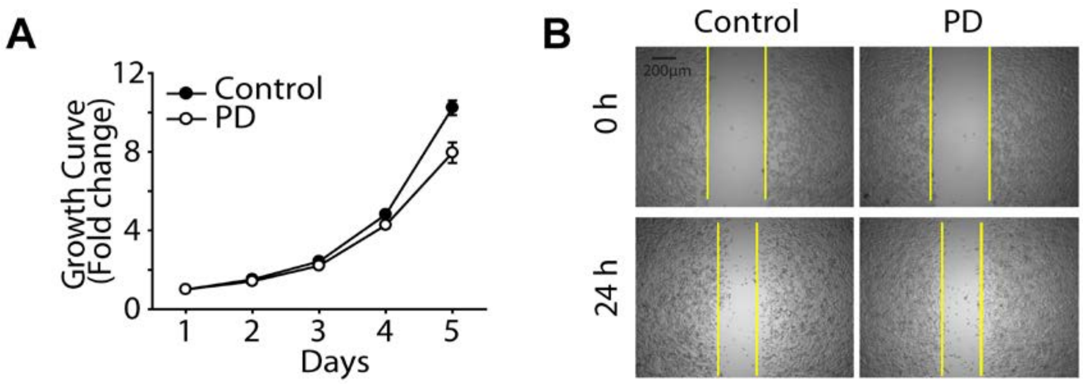

Figure S5. Direct Effects of PD on LLC cells. (A) SRB assay was used to examine the cell proliferation inhibitory activities. (B) Wound-healing assay was used to examine the effect of PD on LLC cells migration.

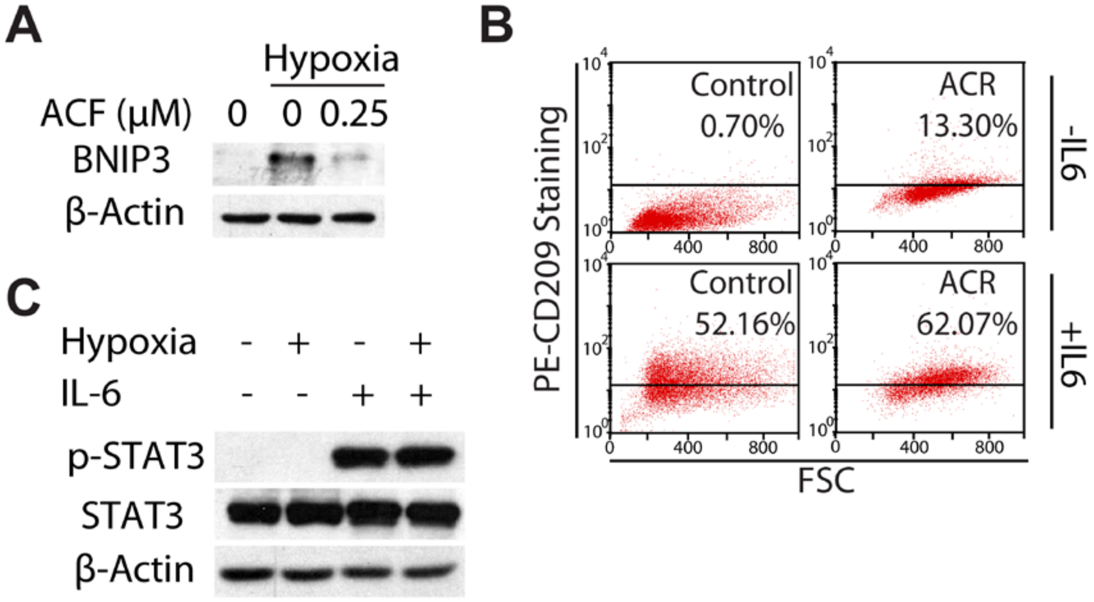

Figure S6. Both HIF activity and STAT3 signaling are not critical events in the M2 macrophage polarization in response to IL6 plus hypoxia. (A) RAW264.7 cells were incubated with acriflavine for 24 h. Then, cells were harvested and lysates were immunoblotted with an anti-BNIP3 antibody. (B) RAW264.7 cells were pretreated with acriflavine before the addition of IL6 and the induction of hypoxic challenge, and flow

cytometric analysis was performed to analyze the expression of CD209. (C) RAW264.7 cells were exposed to normoxia or hypoxia, in the presence or absence of IL6. Proteins were detected by western-blot analysis.

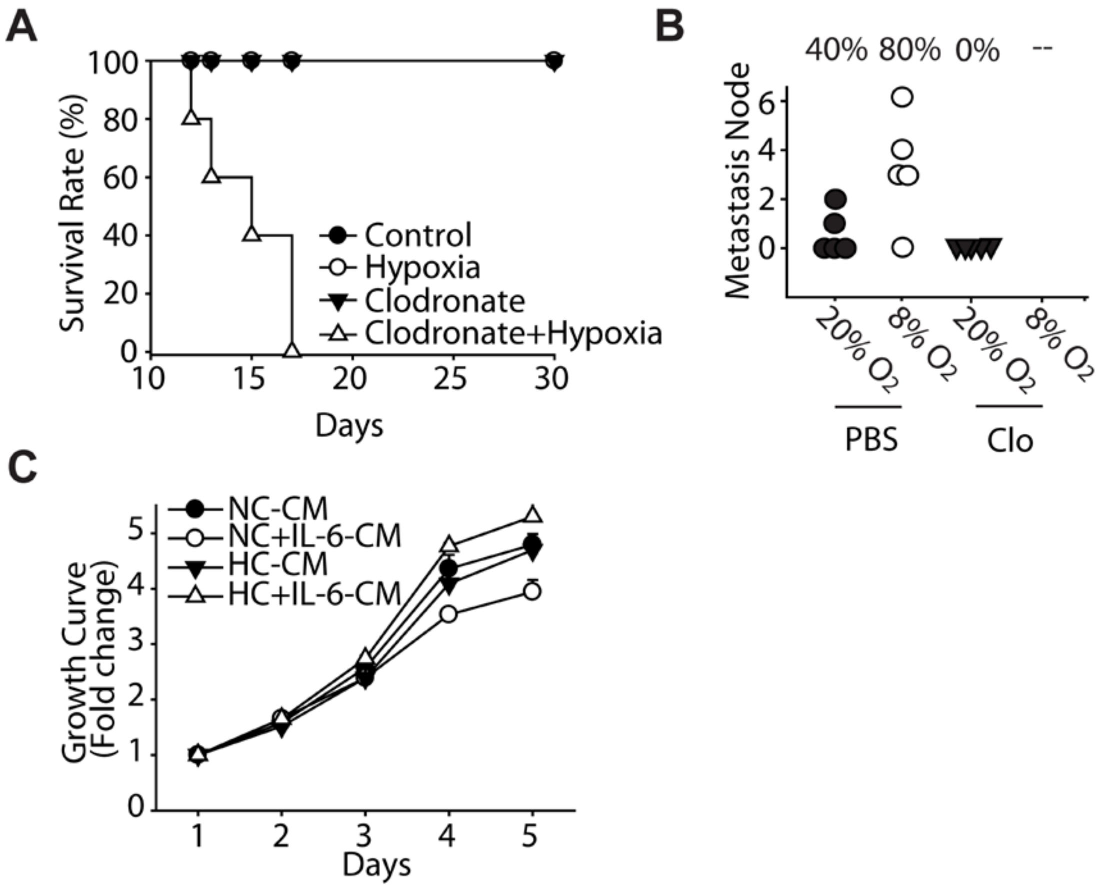

Figure S7. (A) LLC cells were injected subcutaneouly into the armpit of C57BL/6 mice. Mice were assigned to 4 groups: Control group, Clodronate group, Hypoxia group and Clodronate+Hypoxia group (n=5 per group). Within 30 days of inoculation, mice survival was analyzed. (B) Quantitative analysis of lung metastasis nodules (rate). (C) The cell survival was determined in LLC cells treated with indicated macrophage-conditioned mediums for 5 days.
